# JNK and Macroautophagy Activation by Bortezomib Has a Pro-Survival Effect in Primary Effusion Lymphoma Cells

**DOI:** 10.1371/journal.pone.0075965

**Published:** 2013-09-26

**Authors:** Marisa Granato, Roberta Santarelli, Lavinia V. Lotti, Livia Di Renzo, Roberta Gonnella, Alessia Garufi, Pankaj Trivedi, Luigi Frati, Gabriella D’Orazi, Alberto Faggioni, Mara Cirone

**Affiliations:** 1 Department of Experimental Medicine, Istituto Pasteur Fondazione Cenci Bolognetti, La Sapienza University, Rome, Italy; 2 Department of Experimental Oncology, Molecular Oncogenesis Laboratory, “Regina Elena” National Cancer Institute, Rome, Italy; 3 Department of Medical, Oral and Biotechnological Sciences, University “G. d’Annunzio”, Chieti, Italy; Westmead Millennium Institute, University of Sydney, Australia

## Abstract

Understanding the mechanisms of autophagy induction and its role during chemotherapeutic treatments is of fundamental importance in order to manipulate it to improve the outcome of chemotherapy. In particular whether the bortezomib-induced autophagy plays a pro-survival or pro-death role is still controversial. In this study we investigated if bortezomib induced endoplasmic reticulum (ER) stress and activated autophagy in Primary Effusion Lymphoma (PEL) cells and how they influenced cell survival. We found that bortezomib induced up-regulation of the pro-survival and pro-death ER stress molecules BIP and CHOP and activated c-Jun NH2-terminal kinase (JNK), resulting in Bcl-2 phosphorylation and induction of autophagy. JNK and autophagy activation played a pro-survival role in this setting, thus their inhibition increased the bortezomib cytotoxic effect and PARP cleavage in PEL cells. Based on our results we suggest that the combination of bortezomib with JNK or autophagy inhibitors could be exploited to improve the outcome of therapy of this aggressive B cell lymphoma.

## Introduction

Primary Effusion Lymphoma (PEL) is a Kaposi's sarcoma-associated herpesvirus (KSHV)-associated non-Hodgkin’s B-cell lymphoma characterized by poor prognosis because of its aggressive nature and also because of the lack of optimized therapy [1]. We [2] and others [3] have previously shown that bortezomib, a 26S proteasome inhibitor, approved by the Food and Drug Administration (FDA) for the treatment of multiple myeloma (MM) [4], is able to induce apoptosis in PEL cell lines. Bortezomib has been reported to induce the Endoplasmic Reticulum (ER) stress, to activate the unfolded protein response (UPR) and trigger apoptosis and autophagy in several tumor cells [5]. The UPR, required for cell survival in the face of endoplasmic reticulum (ER) stress [6]–[8], is initiated by three ER transmembrane receptors: PKR-like ER kinase (PERK), inositol-requiring enzyme 1 (IRE1), and activating transcription factor 6 (ATF6). PERK phosphorylates the α subunit of eukaryotic initiation factor 2 (eIF2α) to inhibit protein translation [9], IRE1 and ATF6 up-regulate ER chaperon proteins, such as BiP/GRP78 to improve the protein folding activity and increase expression of genes involved in proteasomal degradation by ER-associated protein degradation (ERAD) pathway [10]. The ER stress and the UPR can activate autophagy through IRE1-mediated c-Jun NH2-terminal kinase (JNK) activation and eventually induce apoptosis when cytoprotective functions of the ER are overwhelmed [11].

Macroautophagy (hereafter indicated as autophagy), the process by which a cell “eats” itself, can be a compensatory mechanism of the proteasome inhibition [12], [13], indeed autophagy and ubiquitin proteasome system (UPS) degradation pathways are complementary in the catabolism of unwanted cellular products. Both can degrade misfolded ubiquinated soluble proteins, the difference being that autophagy is able to degrade more substrates such as long-lived proteins, protein aggregates and entire cellular organelles [14], [15].

Autophagy occurs at basal levels in all cells and is activated under metabolic stress, such as nutrient deprivation or when the bioenergetics demand is increased, as in tumor cells. Moreover it can be also induced by the accumulation of intracellular calcium and by ER stress, such as by chemotherapeutic treatments [16]. The process, characterized by the formation of double-membrane vesicles, the autophagosomes, that surrounding aggregated proteins and damaged organelles, fuse with lysosomes where their content is degraded and recycled [17], [18]. Elongation of the autophagosome membrane demands conjugation of MAP1-light chain 3 (LC3) proteins to phosphatidylethanolamine resulting in reduction of the non-lipidated precursor form of the endogenous LC3 (LC3-I) and increase of the lipidated LC3 (LC3-II). The lipidated LC3-II remains associated with autophagosome until fusion with lysosomes and eventually is degraded [19]. Several pharmacologic inhibitors have been used to evaluate the physiological relevance of autophagy in culture cells, including 3-methyladenine (3-MA) which blocks autophagosome formation [20], the lysosomotropic drugs chloroquine (CQ) that raises intralysosomal pH and impairs autophagic protein degradation and bafilomycin A (Baf) which inhibits the vacuolar proton pump (V-H^+^-ATPase) and prevents the proper acidification of lysosomal compartments [21], [22].

To target proteins for autophagic degradation, ubiquitin on modified proteins is recognized and bound by molecules, such as p62/SQSTM1 (p62) which interacts with LC3 to deliver this cargo to autophagosomes [23]. p62, being itself degraded mainly through autophagy, is considered a read-out of the bona-fide autophagic process [24]. _ENREF_26The JNK activation by bortezomib has been reported to induce autophagy by up-regulating Beclin-1 expression or by phosphorylating Bcl-2 [25], [26], disruption of Bcl-2/Beclin 1 complex and releasing Beclin 1 that promotes autophagy [27]. As already mentioned, autophagy is typically a cell survival mechanism during proteasome inhibition [28], [29], however in tumors such as multiple myeloma (MM), characterized by basal high ER stress and intense UPR, bortezomib-induced autophagy has been shown to contribute to cell death [30]. As PEL resembles MM in its characterization of high basal protein synthesis [31], in this study we aimed at investigating the impact of bortezomib on ER stress and autophagy and their role in PEL cell survival. We found that bortezomib induced ER stress and autophagy in PEL cell lines, with up-regulation of ER stress-dependent pro-survival and pro-apoptotic molecules such as BiP and CHOP. It also activated JNK, that positively regulates autophagy. Autophagy and JNK activation had a pro-survival effect, thus their inhibition increased the bortezomib cytotoxicity in PEL cells.

## Materials and Methods

### Cells and reagents

The BC3, BCBL1, JSC1 and BC1 PEL cell lines (ATCC) were cultured in RPMI 1640 10% Fetal Calf Serum (FBS) (Euroclone), glutamine and streptomycin (100 µg/ml) and penicillin (100U/ml) in 5% CO_2_ at 37°C. The proteasome inhibitor bortezomib (Velcade) was purchased from Millennium Pharmaceutical Inc. 3-methyladenine (3-MA), Bafilomycin A (Baf), the JNK inhibitor SP600125 and ERK inhibitor PD98059 were purchased by Sigma Aldrich.

We used the following primary antibodies: rabbit polyclonal anti-LC3 (1∶1000) (Novus Biologicals), mouse monoclonal anti-p62 (1∶1000) (BD Transduction Laboratories), rabbit polyclonal anti-ATG5 (1∶1000) (Novus Biologicals), rabbit polyclonal anti-BiP (1∶1000) (Cell Signaling), mouse monoclonal anti-CHOP (1∶1000) (Cell Signaling), rabbit polyclonal anti-IRE1α (1∶1000) (Cell Signaling), rabbit polyclonal anti-Phospho-JNK (pJNK) (1∶500) (Thr183/Tyr185) (Cell Signaling), rabbit polyclonal anti-t-JNK (t-JNK) (1∶500) (Cell Signaling), rabbit polyclonal anti-Phospho-Bcl2 (S70) (1∶500) (Cell Signaling), rabbit polyclonal anti-t-Bcl2 (t-Bcl2) (1∶500) (Cell Signaling) and rabbit polyclonal anti-PARP (Poly-ADP-ribose polymerase) p85 Fragment (1∶1000) (Promega). A monoclonal mouse anti-α-tubulin (1∶1000) or anti-β-actin (1∶10000) (Sigma Aldrich) were used as marker of equal loading. All the primary antibodies used in this study, were diluted in a PBS-0.1% Tween20 solution containing 3% of BSA.

### Western blot analyses

Cells (5×10^5^) were washed twice with PBS and lysed in a modified RIPA buffer containing 150 mM NaCl, 1% NP-40, 50 mM Tris-HCl (pH 8), 0.5% deoxycholic acid, 0.1% SDS, 1% Triton X-100, protease and phosphatase inhibitors. The lysates were subjected to electrophoresis on 4–12% NuPage Bis-Tris gels (Life Technologies) and transferred to PVDF membranes (Thermo Scientific). The membranes were blocked and probed with specific antibodies and blots were developed using ECL Blotting Substrate (Thermo Scientific).

### Electron Microscopy (EM) analysis

Cells were fixed in 2% glutaraldehyde in PBS for 24 h at 4°C, post-fixed in 1% OsO4 for 2 h and stained for 1 h in 1% aqueous uranyl-acetate. The samples were then dehydrated with graded acetones and embedded in Epon-812 (Electron Microscopy Science, Società Italiana Chimici). One micron thick sections were cut, stained with 1% methylene blue and viewed by light microscopy to select representative areas. Ultrathin sections were cut with a Reichert ultramicrotome, counterstained with uranyl-acetate and lead citrate, and examined with a Philips CM10 transmission electron microscope (FEI, Eindhoven, The Netherlands).

### RNA extraction and reverse transcription (RT)-PCR analysis

Cells were harvested in TRIzol Reagent (Invitrogen) and total RNA was isolated following the manufacturer’s instruction, as previously described [32]. cDNA was syntesized from 2 µg of total RNA with MuLV reverse transcriptase kit (Applied Biosystems). Semi-quantitative RT-PCR was carried out by using Hot-Master Taq polymerase (Eppendorf) with 2 µl cDNA reaction and genes specific oligonucleotides under conditions of linear amplification. PCR was performed in duplicate in two different sets of cDNA. PCR products were run on a 3% agarose gel and visualized by ethidium bromide staining using UV light. The housekeeping α-actin was used as internal standard. Densitometric analysis was applied to quantify specific mRNA levels compared to internal standard.

### Cell proliferation

PEL cells were plated in 12-well plates at a density of 5×10^5^ cells/ml and treated with bortezomib (20 nM), 3-methyladenine (5 mM), SP600125 (10 µM), alone or in combinations or cells were knocked down for ATG5 before bortezomib treatment for the indicated time. DMSO was used as control. After treatment, cells were collected and counted by trypan-blue exclusion using a Neubauer hemocytometer. The percentage of cell viability, as blue/total cells, was assayed by scoring 200 cells per well three times.

### Transfection and plasmids

PEL cells were transfected by using the polymer-based DNA transfection reagent jetPEI™(Polyplus-transfection), according to the manufacture’s specifications. The amount of plasmid DNA was equalized in each sample by supplementing with empty vector and transfection efficiency was visualized with the use of a co-transfected GFP expression vector. The expression plasmid used was the nonphosphorylatable (competitive inhibitor) HA-JNK-APF (DN-JNK) [33] (Kindly provided by Lynn E. Heasley, University of Colorado, Aurora, CO).

### Knockdown by small interfering RNA of ATG5

The knockdown of ATG5 was performed by specific small interfering RNA according to the manufacturer’s’ instructions (Santa Cruz).

Briefly, the day before transfection, 3×10^5^ PEL cells were seeded in 24 well culture plate in RPMI medium w/o antibiotics. Then, 60 pmoli of siRNA duplex and 6 µl of siRNA Transfection Reagent (Santa Cruz) were used and bortezomib (20 nM) was added after 24-48 hrs. To evaluate the transfection efficiency a fluorescein conjugated control siRNA (Santa Cruz) was used.

## Results

### Bortezomib induces ER stress and JNK activation in PEL cell lines

**Figure 1:**
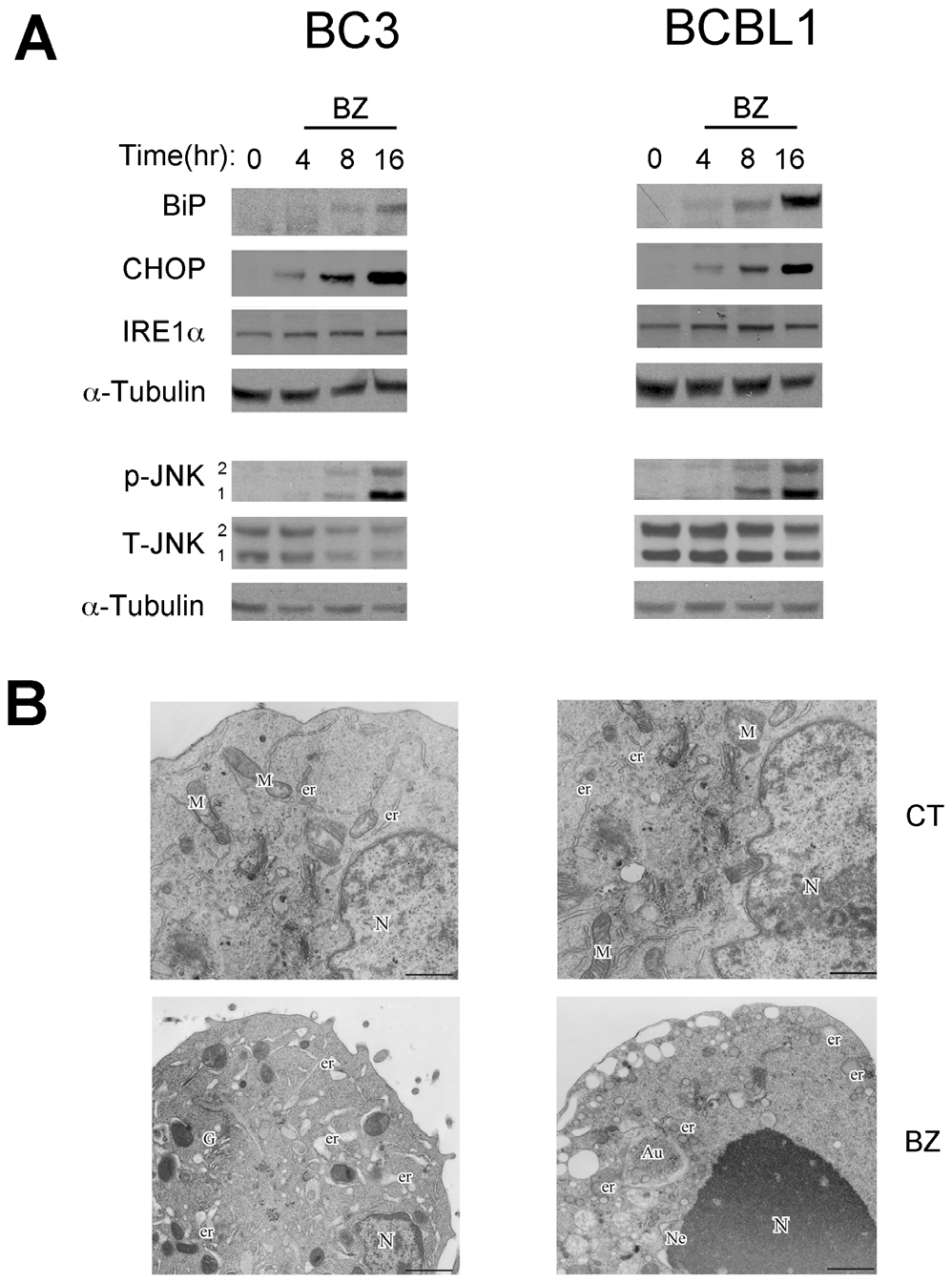
Bortezomib treatment induces ER stress in PEL cell lines. (A) Time course assay was performed on BC3 and BCBL1 cells treated with 20 nM of bortezomib (BZ) for 0, 4, 8, 16 hrs. Total cell lysates were prepared and immunoblotted with anti-BiP, anti-CHOP, anti IRE1α, anti-phospho-JNK (p-JNK 1/2) and t-JNK antibodies. Anti-tubulin was assayed as protein loading control. (B) Electron microscopy (EM) analysis was performed on BC3 and BCBL1 cells treated with Bortezomib (20 nM) for 16 hrs (BZ). Cells showed several signs of ER stress compared to untreated control (CT). More apoptotic features such as nuclear (N) condensation were observed in BCBL1. Er (Endoplasmic reticulum), G (Golgi Apparatus), N (Nucleus), Au (Autophagosomes), Ne (Nuclear Envelope), M (Mitochondria). Bars: 1 µm. Results are representative of three independent experiments.

Bortezomib inhibition of cytosolic proteasome can induce ER stress, due to the unfolded protein accumulation, in several cancer cells, especially those characterized by high level of protein synthesis [34]. Therefore, we aimed at investigating if bortezomib induced ER stress in PEL cell lines. We found a time-dependent increase of both pro-survival and pro-death ER stress-related molecules, respectively, BiP/GRP78 (BiP) and C/EBP homologous (CHOP), both in BC3 and BCBL1 cell lines (Figure 1A). Following ER stress, the activation of some UPR genes can, until a certain limit, hamper cell death by limiting unfolded protein accumulation and mitigating the ER stress [35]. In both PEL cell lines, beside the induction of BiP, we found that proteasome inhibition by bortezomib determined a slight IRE1α upregulation (Figure 1A). Finally, JNK phosphorylation was analyzed, since IRE1 can activate, among other molecules, JNK that, in turn, may regulate cell survival [36]. We found JNK1/2 phosphorylation by bortezomib in both BC3 and BCBL1 cells (Figure 1A). ER stress was also evaluated by electron microscopy (EM) analysis. BC3 and BCBL1 cells treated with bortezomib showed severe signs of ER stress, such as dilatation of endoplasmic reticulum (Figure 1B-BZ) that were not observed on control cells (Figure 1B-CT).

### Bortezomib induces autophagy in PEL cells

**Figure 2:**
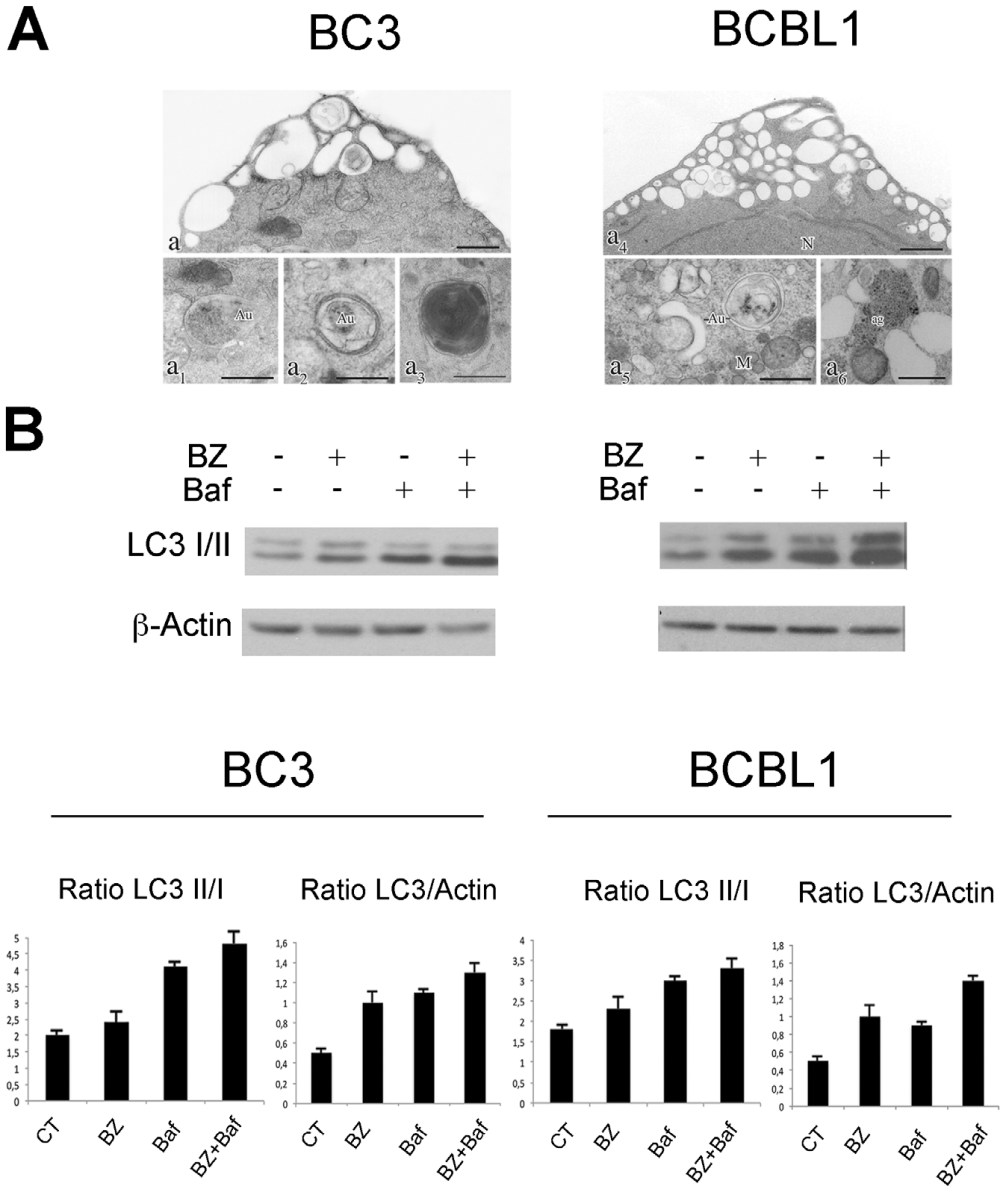
Bortezomib induces autophagy in PEL cells. (A) Electron microscopy (EM) on BC3 and BCBL1 cells treated with 20 nM of bortezomib after 16 hrs shows pronounced cell vacuolization (A a, a_4_) with autophagosome membrane-bound compartment in different stage of maturation (A a_1,_ a_2_, a_4,_ a_5_). Small intracellular structures consistent with aggresomes were evident in BCLB1 cells (A a_6_). N (Nucleus), Au (Autophagosomes), M (Mitochondria), ag (aggresomes). Bars 1 µm. Results are representative of three independent experiments. (B) PEL cells were treated with bortezomib (20 nM), bafilomycin A (Baf) (10 nM) or combination of both. The western blot analysis was performed to detect the two isoforms of LC3 protein. The histograms indicate LC3-II/I and LC3-II/Actin ratio based on densitometric analysis (mean ± the standard deviation, n = 3 experiments). An antibody against β-actin was used as loading control.

As bortezomib-mediated block of proteasome may promote autophagy in some tumor cells, [26] we investigated the effect of bortezomib on PEL cell autophagy. For this purpose we used several different approaches according to [37]. We first evaluated the presence of autophagosomes by EM analysis and found a strong vacuolation (a, a_4_) as well as several double membrane vacuoles (autophagosomes) in different stages of maturation in BC3 and BCBL1 PEL cell lines (a_1,_ a_2,_ a_5_) upon 16 hours of bortezomib treatment (Figure 2A)_._ In BC3 cells some multilamellar bodies were also observed (a_3_) while in BCBL1 cells, beside the more numerous apoptotic features, we observed many “aggresomes” (a_6_), likely as a result of a greater accumulation of aggregated unfolded proteins [38].

We next measured the LC3 protein levels by Western blot and found an increase of LC3-II in both cell lines, upon bortezomib treatment, as also evidenced by densitometric analysis of LC3-II/I and LC3-II/Actin ratios (Figure 2B), suggesting an impact of bortezomib on PEL cell autophagy. The conversion of LC3-I in LC3-II occurs during autophagy, however it is also true that this molecule is eventually degraded in a complete autophagic flux [37]. Hence to further clarify if bortezomib was promoting or blocking the basal autophagy in PEL cells, we compared the levels of LC3-II induced by bortezomib with bafilomycin A1 (Baf) to the single treatment with Baf, an inhibitor of the lysosome acidification, that blocks autophagy at its final steps, as previously reported [29]. The results indicate that the level of LC3-II further increased in cells treated with the combination of bortezomib with Baf in comparison to the single treatments (Figure 2B), also evidenced by densitometric analysis, suggesting that an efficient autophagy was induced by bortezomib in PEL cells.

### Bortezomib-induced autophagy is positively regulated by JNK

**Figure 3:**
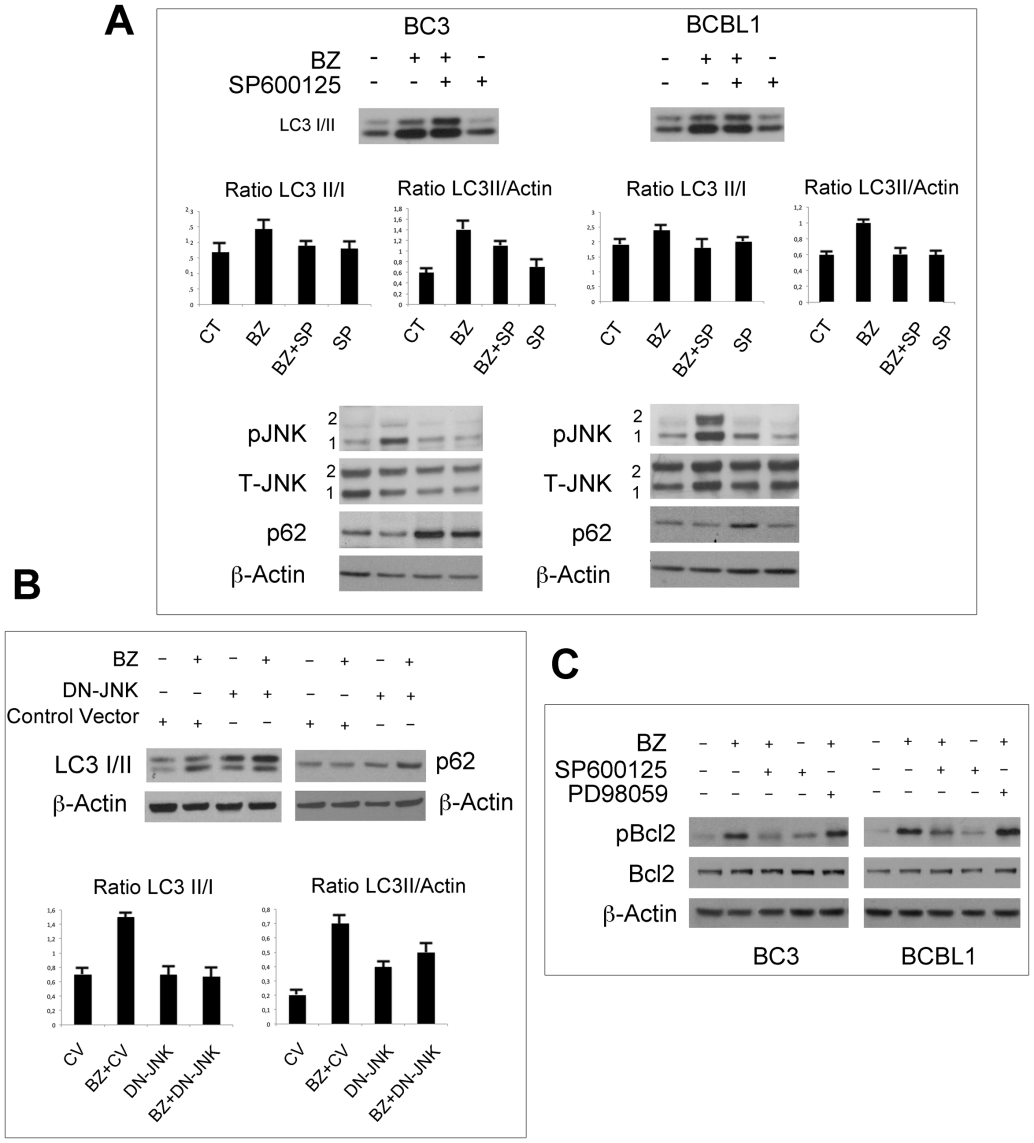
Bortezomib induces autophagy in JNK-dependent manner. (A) BC3 and BCBL1 cells were treated with bortezomib (20 nM for 16 hrs) with or without pre-treatment with JNK inhibitor (SP600125) (20 µM). Total cell lysates were prepared and immunoblotted with the following antibodies: anti-LC3, anti-pJNK, anti-T-JNK and anti-p62. Anti-β-actin was used as loading control. The histograms indicate LC3-II/I and LC3-II/Actin ratio based on densitometric analysis (mean ± the standard deviation, n = 3 experiments). (B) BC3 cells were transfected with DN-JNK expression vector or with an control empty vector (CV) and after 16 hrs treated with bortezomib (20 nM) for an additional 16 hrs. Total cell lysates were prepared and immunoblotted with anti-LC3 and p62 antibodies. β-actin was used as internal control. The histograms indicate LC3-II/I and LC3-II/Actin ratio based on densitometric analysis (mean ± the standard deviation, n = 3 experiments). (C) BC3 and BCBL1 cells were treated with Bortezomib (20 nM) alone or in combination with JNK inhibitor (SP600125) (20 µM) or ERK inhibitor (PD98059) (10 µM) for 16 hrs. A western blotting was performed using the following antibodies: anti-pBcl2(S70) and anti-total Bcl2. β-actin was used as loading control.

To evaluate the role of JNK1 and JNK2 activation in the bortezomib-induced autophagy we analyzed the level of the LC3-I and LC3-II in PEL cells treated with bortezomib for 16 hours with or without pretreatment with SP600125, a JNK1 and JNK2 specific inhibitor, by western-blot analysis. The results show a reduction of the LC3-II/I ratio in the presence of SP600125, suggesting that a possible autophagic block was occurring following JNK inhibition (Figure 3A). Concomitantly we verified that the bortezomib-induced JNK1 and JNK2 phosphorylation was effectively inhibited by its specific inhibitor SP600125 (Figure 3A). Next p62 was evaluated in the presence or in the absence of SP600125, since p62 decrease is considered a read-out of a complete autophagic process. Bortezomib treatment caused a degradation of p62 in BC3 and in BCBL1 indicating a complete autophagic flux; however, pre-treatment with JNK inhibitor SP600125 impaired bortezomib-induced p62 degradation, leading to p62 accumulation (Figure 3A) and therefore to a block of autophagy.

As an additional approach of JNK1 and JNK2 inhibition, BC3 cells were transfected with a nonphosphorylatable (competitive inhibitor) HA-JNK-APF plasmid (DN-JNK) for 24 hours before bortezomib treatment [33] and found a decrease of LC3-II/I ratio and an increase of p62 compared to cells transfected with control vector (Figure 3B), in agreement with the results obtained with SP600125 (Figure 3A). Since it has been previously reported that JNK-mediated Bcl2 phosphorylation regulates starvation-induced autophagy [39], we analyzed if bortezomib-induced JNK phosphorylation was promoting autophagy through the phosphorylation of Bcl2. We found that JNK inhibitor SP600125 efficiently counteracted Bcl2 phosphorylation, while the use of PD98059 ERK inhibitor did not (Figure 3C), suggesting that bortezomib-mediated JNK activation is promoting autophagy likely by inducing Bcl2 phosphorylation.

### Inhibition of autophagy and JNK increase the bortezomib anti-proliferative effect

**Figure 4:**
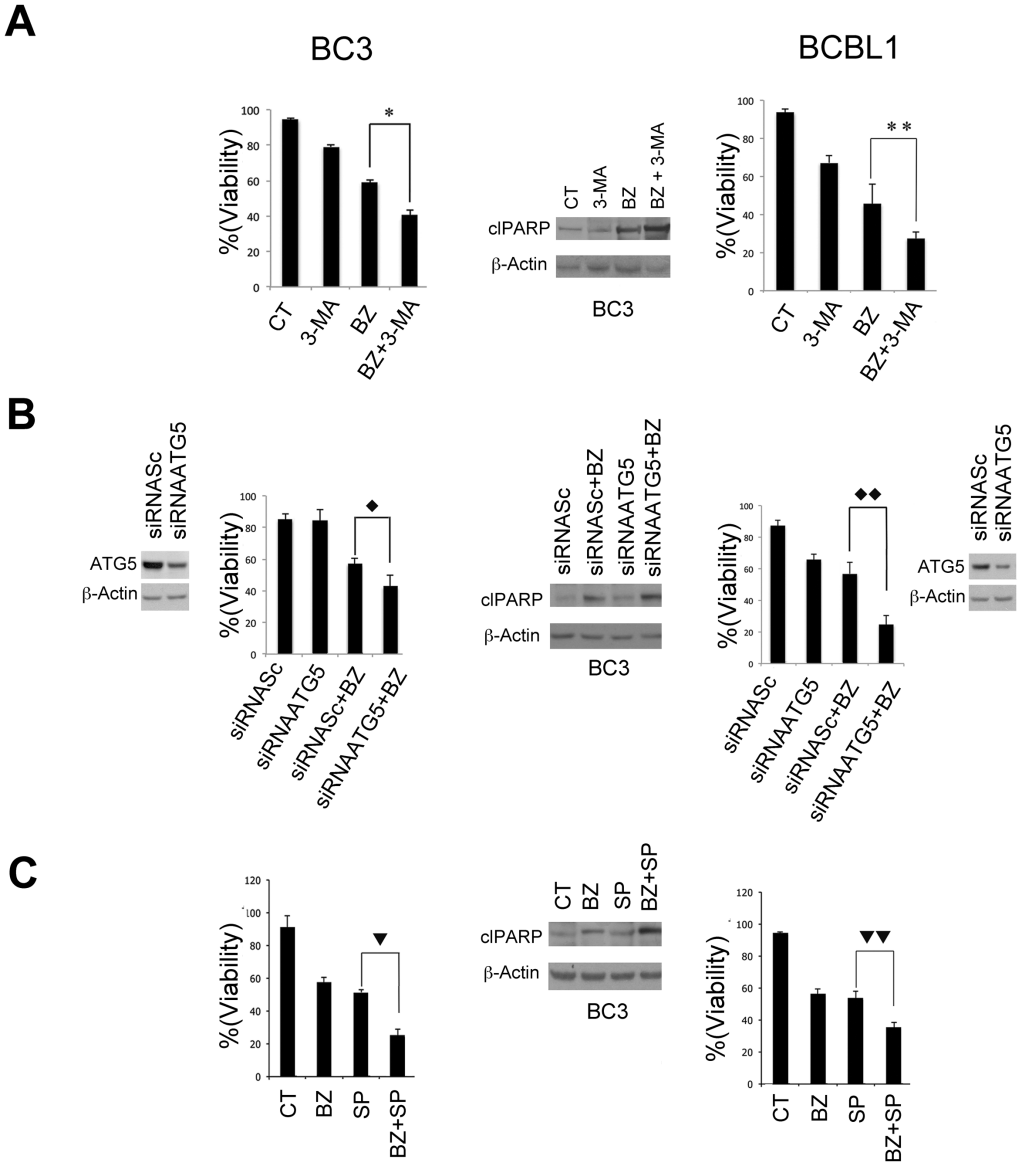
Inhibition of autophagy and JNK increases the Bortezomib anti-proliferative effect in BC3 and BCBL1 PEL cells. (A) Viability assay evaluated by trypan blue exclusion was performed in BC3 (left panel) and BCBL1 (right panel) cells treated with bortezomib (20 nM) or 3-MA (5 mM) alone or in combination for 16 hrs. Mean ± the standard deviation was also indicated (n = 3 experiments). * p-value  =  0.05, ** p-value  =  0.02. Western blotting analysis was performed on BC3 cells to evaluate the expression of cleaved (cl) PARP p85 fragment (middle panel). β-actin was used as loading control. (B) BC3 and BCBL1 cells were transfected with ATG5 siRNA or scramble siRNA (siRNASc), and than a western blot was performed with the anti-ATG5 antibody. β-actin was used as loading control. Viability assay evaluated by trypan blue exclusion was performed in BC3 and BCBL1 cells ATG5 or scramble-knocked down upon bortezomib treatment (20 nM) for 16 hrs. Mean ± the standard deviation was indicated (n = 3 experiments). ♦ p-value  =  0.02, ♦♦ p-value  =  0.03. Western blotting analysis was also performed on BC3 cells to evaluate the expression of PARP p85 fragment (cl PARP) and β-actin was used as internal control. (C) Cells viability assay on BC3 and BCBL1 cells treated with bortezomib (20 nM) and SP600125 (20 µM) alone or in combination for 16 hrs. The percentage of live cells was evaluated by trypan blue exclusion assay. Mean ± the standard deviation was indicated (n = 3 experiments). p-value  =  0.01, p-value  =  0.01. Western blotting analysis was performed on BC3 cells to evaluate the expression of PARP p85 fragment (cl PARP) and β-actin was used as loading control.

We next evaluated the role of the bortezomib-induced autophagy on PEL cell survival. To this aim, we first combined bortezomib treatment with 3-MA, an inhibitor of the initial step of autophagy [18]. The results obtained show that 3-MA increased bortezomib-antiproliferative effect in both BC3 and BCBL1 cell lines (Figure 4A) and also increased PARP cleavage (cl PARP) (Figure 4A), a marker of an apoptotic cell death.

Conversely the combination of bortezomib with the autophagy inducer rapamycin reduced its cytotoxic effect (data not shown). In order to exert a more specific effect on the autophagy inhibition, we performed a knocking-down of ATG5 [29], an essential autophagic gene. According to the above results, we found that the ATG5 knocking-down also potentiated the bortezomib cytotoxicity and PARP-cleavage (cl PARP) compared to the scramble-transfected cells (Figure 4B). The ATG5 knock-down was verified by comparing the expression of ATG5 in BC3 and BCBL1 cells transfected with ATG5 (siRNAATG5) or Scramble siRNA (siRNASc), by western blotting (Figure 4B). We then investigated if JNK activation, shown to promote autophagy in PEL cells during bortezomib treatment, had a pro-survival effect in this setting. To this purpose we inhibited JNK by using SP600125 before bortezomib treatment and found that JNK inhibitor potentiated both the cytotoxic effect of bortezomib in PEL cells and PARP-cleavage (cl PARP) (Figure 4C).

### JNK and autophagy are activated as pro-survival effects also in PEL cells that harbor EBV infection together with KSHV

**Figure 5:**
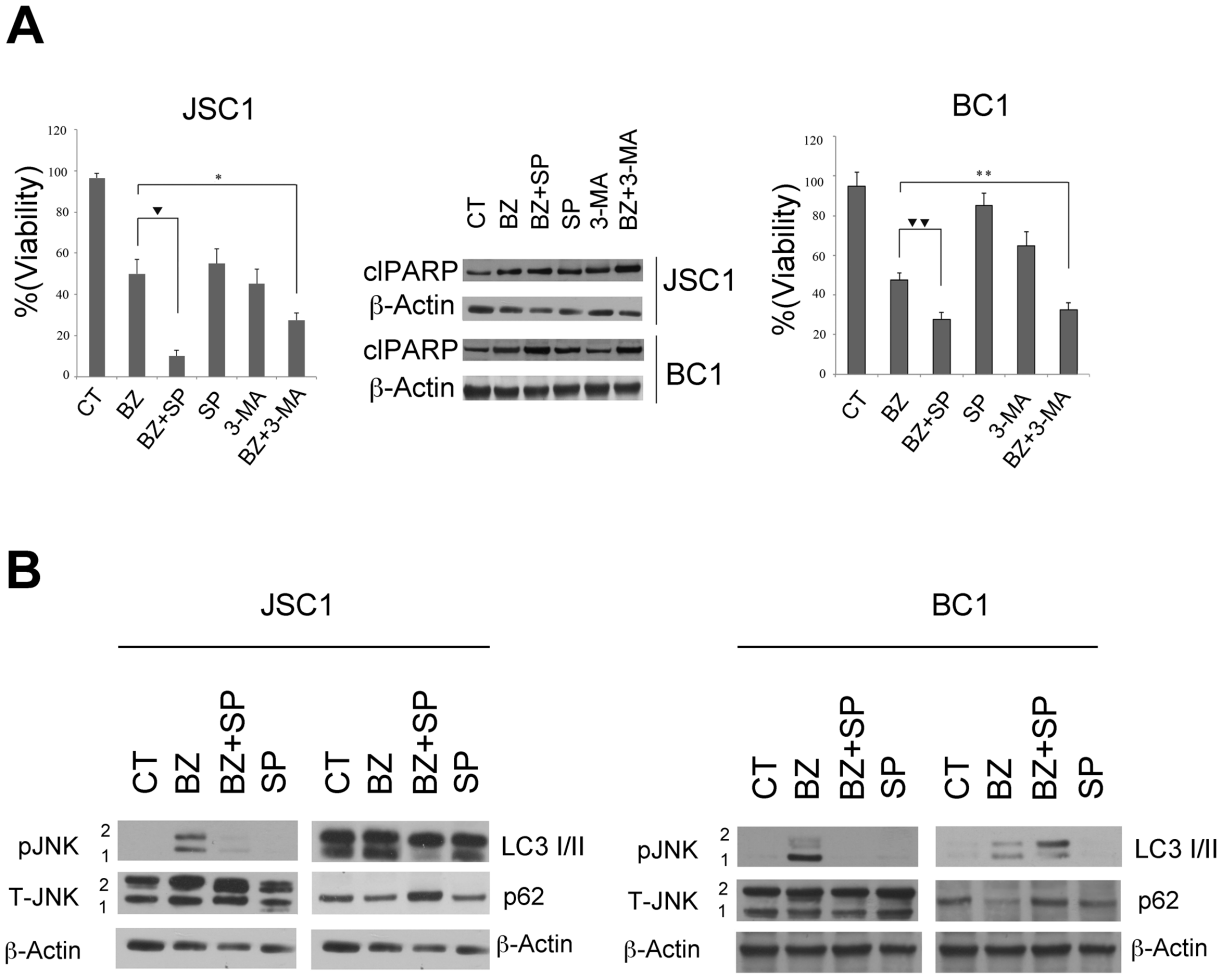
Inhibition of autophagy with 3-MA or with JNK inhibitor increases the Bortezomib anti-proliferative effect in JSC1 and BC1 PEL cells. (A) Viability assay evaluated by trypan blue exclusion was performed in JSC1 (left panel) and BC1 (right panel) cells treated with bortezomib (20 nM) or 3-MA (5 mM) or SP600125 (20 µM) alone or in combination for 16 hrs. Mean ± the standard deviation was also indicated (n = 3 experiments). * p-value  =  0.05, ** p-value  =  0.05, ♦p-value  =  0.01, ♦♦p-value  =  0.02. Western blotting analysis was performed on JSC1 and BC1 cells to evaluate the expression of cleaved (cl) PARP p85 fragment. β-actin was used as loading control (middle panel). JSC1 and BC1 cells were treated with bortezomib (20 nM for 16 hrs) with or without pre-treatment with JNK inhibitor (SP600125) (20 µM). Total cell lysates were prepared and immunoblotted with the following antibodies: anti-pJNK, anti-T-JNK, anti LC3I/II and anti p62. Anti-β-actin was used as loading control.

Since PEL can be, in some patients, dually infected with KSHV and Epstein-Barr virus (EBV), we extended our study to two other PEL cell lines, namely JSC1 and BC1, that have this characteristic. We found that bortezomib had an higher cytotoxic effect also in these cells when combined with the autophagy or JNK inhibitors, respectively 3-MA and SP600125 (Figure 5A). This effect was also evidenced by the increase of PARP cleavage (cl-PARP) in the cells treated with the combination therapies (Figure 5A). We finally investigated if JNK inhibition by SP600125 correlated with a block of the pro-survival autophagy induced by bortezomib, as for BC3 and BCBL1 cells and found that indeed it reduced the LC3-II formation and increased p62 also in JSC1 and BC1 cells (Figure 5B).

Altogether these results indicate that JNK and autophagy are activated during bortezomib treatment and played a pro-survival role in PEL cell lines either single infected with KSHV either dually infected with KSHV and EBV.

## Discussion

Primary Effusion Lymphoma (PEL) is a KSHV-associated non-Hodgkin’s B-cell lymphoma characterized by poor prognosis because of its aggressive nature and also because of the lack of optimized therapy [1]. Thus, new therapeutic targets and strategies are needed for this disease. We have recently shown that bortezomib induces apoptosis in PEL cell lines [2] and in this paper we investigated which strategies could be used to improve its cytotoxic effect in these cells. An emerging field of thought suggests that the cellular process of autophagy may represent a novel therapeutic target, expecially if it is induced during chemotherapeutic treatment as survival mechanism. Our studies show that a complete autophagic flux was induced by bortezomib in PEL cells as demonstrated by the increase of LC3-II when it is combined with bafilomycin (Baf) and by p62/SQSTM1 (p62) protein degradation that is considered a read-out of the bona-fide autophagic process [24].

Autophagy is usually a pro-survival mechanism [35], but it can also contribute to cell death in tumors such as multiple myeloma (MM) that shares several features with PEL [4], [40]. We found that bortezomib induced a pro-survival autophagy in PEL cells, thus its antiproliferative effect can be potentiated by its combination with pharmacological autophagy inhibitors or by ATG5 knock-down. Moreover the combination of bortezomib with an autophagy inducer, such as rapamycin, was able to partially reduce the bortezomib cytotoxicity in PEL cells (data not shown). Autophagy correlated with bortezomib-induced endoplasmic reticulum (ER) stress and JNK1 and JNK2 activation in PEL cells. JNK activation can induce autophagy in several ways, including phosphorylation of Bcl2 and impairment of Bcl2/beclin1 interaction [27], up-regulation of beclin [25], dephosphorylation of mTOR [41] or up-regulation of p62 [40]. It has been previously shown that bortezomib activates JNK [40], [42] although a link between its activation and bortezomib-induced autophagy has not been clearly elucidated yet. In our studies we observed that bortezomib-mediated JNK activation induced Bcl2 phosphorylation, specifically inhibited by SP600125, suggesting that this may represent the main mechanism of autophagy induction in PEL cells. According to that, JNK inhibition by SP600125 reduced the LC3-II/I ratio, impaired p62 protein degradation and improved the bortezomib cytotoxicity, indicating the pro-survival effect of bortezomib-mediated JNK activation and autophagy induction.

In summary, PEL cells, harboring only KSHV or KSHV and EBV, treated with the proteasome inhibitor bortezomib led to JNK1 and JNK2 activation and to a complete autophagic flux. JNK and autophagy inhibition strongly improved the bortezomib-induced antiproliferative effect in these cells. On the basis of these evidences, our results suggest that the combination of bortezomib with autophagy or JNK inhibitors might be exploited to improve the antitumor therapy in PEL.
